# Multi‐Bioinspired Functional Conductive Hydrogel Patches for Wound Healing Management

**DOI:** 10.1002/advs.202301479

**Published:** 2023-06-27

**Authors:** Wenzhao Li, Yunru Yu, Rongkang Huang, Xiaocheng Wang, Puxiang Lai, Kai Chen, Luoran Shang, Yuanjin Zhao

**Affiliations:** ^1^ Department of Rheumatology and Immunology, Nanjing Drum Tower Hospital School of Biological Science and Medical Engineering Southeast University Nanjing 210096 China; ^2^ Oujiang Laboratory (Zhejiang Lab for Regenerative Medicine, Vision and Brain Health), Wenzhou Institute University of Chinese Academy of Sciences Wenzhou Zhejiang 325001 China; ^3^ Department of Biomedical Engineering The Hong Kong Polytechnic University Hong Kong SAR 999077 China; ^4^ Department of General Surgery and Provincial Key Laboratory of Colorectal and Pelvic Floor Diseases, Guangdong Institute of Gastroenterology The Sixth Affiliated Hospital Sun Yat‐sen University Guangdong 510655 China; ^5^ Department of Orthopedics, Shanghai Changhai Hospital Naval Medical University Shanghai 200433 China; ^6^ Shanghai Xuhui Central Hospital, Zhongshan‐Xuhui Hospital, and the Shanghai Key Laboratory of Medical Epigenetics, the International Co‐laboratory of Medical Epigenetics and Metabolism (Ministry of Science and Technology), Institutes of Biomedical Sciences Fudan University Shanghai 200433 China

**Keywords:** actuator, bio‐inspired, flexible electronics, hydrogel, photo‐response, wound healing

## Abstract

Many hydrogel patches are developed to solve the pervasive and severe challenge of complex wound healing, while most of them still lack satisfactory controllability and comprehensive functionality. Herein, inspired by multiple creatures, including octopuses and snails, a novel muti‐functional hydrogel patch is presented with controlled adhesion, antibacterial, drug release features, and multiple monitoring functions for intelligent wound healing management. The patch with micro suction‐cup actuator array and a tensile backing layer is composed of tannin grafted gelatin, Ag‐tannin nanoparticles, polyacrylamide (PAAm) and poly(*N*‐isopropylacrylamide) (PNIPAm). In virtue of the photothermal gel‐sol transition of tannin grafted gelatin and Ag‐tannin nanoparticles, the patches exert a dual anti‐microbial effect and temperature‐sensitive snail mucus‐like features. In addition, as the “suction‐cups” consisting of thermal responsive PNIPAm can undergo a contract‐relax transformation, the medical patches can adhere to the objects reversibly and responsively, and release their loaded vascular endothelial growth factor (VEGF) controllably for wound healing. More attractively, benefiting from their fatigue resistance, self‐healing ability of the tensile double network hydrogel, and electrical conductivity of Ag‐tannin nanoparticles, the proposed patches can report multiple wound physiology parameters sensitively and continuously. Thus, it is believed that this multi‐bioinspired patch has immense potential for future wound healing management.

## Graphical abstract

Inspired by octopuses and snails, a multifaceted hydrogel patch is developed with NIR controlled adhesion, drug release, antibacterial features, and multiple monitoring function for wound healing intelligent management.

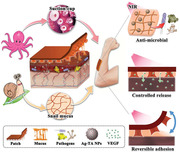

## 1 Introduction

The healing of complex wounds is a pervasive and severe challenge in the medical field. If not handled properly, it will cause inflammation, infection, and even death.^[^ ^1^, ^2^, ^3^, ^4^, ^5^ ^]^ Hydrogel patches possess immense potential due to their unique soft matrix, high water content, excellent biocompatibility, and ease of functionalization.^[^ ^6^, ^7^, ^8^, ^9^, ^10^ ^]^ By integrating interdisciplinary advantages, previously‐reported hydrogel patches have been endowed with various functions including adhesive behaviors, anti‐microbial activity, sustained drug release, and so on.^[^ ^11^, ^12^, ^13^, ^14^, ^15^ ^]^ Although with many progresses, most of these hydrogel patches still have some deficiencies for complex wounds in practice. Generally, the adhesion of common patches is irreversible or unfirm, resulting in uncontrolled adhering and peeling behavior.^[^ ^16^, ^17^ ^]^ In addition, functions such as anti‐bacterial and controlled drug release are passive, which cannot respond flexibly to complex conditions.^[^ ^18^, ^19^ ^]^ Moreover, these patches tend to lack the ability to monitor the state of the wound continuously and effectively.^[^ ^20^, ^21^ ^]^ Thus, a new hydrogel patch with the distinctive functions of intelligently managing wound healing is still urgently needed.

**Scheme 1:**
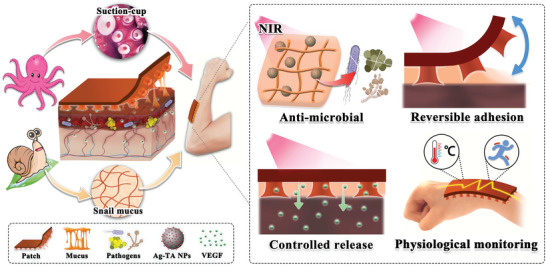
Schematic diagram of multi‐bioinspired functional hydrogel patches for wound healing management. Inspired by the octopus, the patch has a micro suction‐cup array on its backing layer; inspired by the snail, it has mucus filling in the interfaces and gaps. The anti‐microbial, controlled release, and reversible adhesion functions of the patch all can be controlled by near‐infrared (NIR) irradiation, which facilitates intelligent wound healing management.

Here, inspired by multiple creatures, such as octopuses and snails, we present a novel hydrogel patch with controlled adhesion, antimicrobial, drug release, and flexible electronic functions for wound healing management, as illustrated in **Scheme** **1**. In nature, octopus achieves reversible physical adhesion by controlling the movement of their suction‐cups,^[^ ^22^, ^23^, ^24^ ^]^ while snails consolidate their chemical adhesion through dissipation and entanglement of sticky macromolecules in mucus.^[^ ^25^, ^26^ ^]^ Inspired by these natural creatures, various artificial suction‐cups, adhesive grafted polymers, and dissipative double network (DN) hydrogels have been widely investigated.^[^ ^16^, ^17^, ^27^, ^28^ ^]^ Some of these adhesive polymers and hydrogels can be constructed by means of tannin, a plant‐derived small molecule.^[^ ^29^ ^]^ Especially, the Ag‐tannin nanoparticles can endow materials with excellent photothermal, antibacterial, and electrical conductivity.^[^ ^30^, ^31^ ^]^ Despite their great functionalization potential and excellent suitability for the needs of wound repair management, these materials have not yet been integrated into medical patches.

In this paper, we fabricated the desired medical patches by using a mixture of tannin grafted gelatin, Ag‐tannin nanoparticles, polyacrylamide (PAAm) and Poly(N‐isopropylacrylamide) (PNIPAm) to form mucus, DN hydrogel with a micro suction‐cup actuator array and a tensile backing layer. Owing to the photothermal effect of Ag‐tannin nanoparticles and the gel‐sol transition of gelatin, the resultant materials were imparted with a dual anti‐microbial effect, as well as a temperature‐sensitive snail‐mucus‐like feature. In contrast, the “suction‐cups” could perform a contract‐relax transform by controlling the shrinking and swelling of their PNIPAm components. Thus, the medical patches could achieve the function of responsive reversible adhesion. Meanwhile, with their gelatin solation and PNIPAm contraction, the loaded vascular endothelial growth factor (VEGF) could be controllably released from the patches for wound healing through multiple mechanisms, including epithelialization, collagen deposition, and angiogenesis.^[^ ^32^ ^]^ In addition, due to their fatigue resistance, self‐healing ability of tensile DN hydrogel, and electrical conductivity of Ag‐tannin nanoparticles, the resultant patches could monitor wound physiology such as temperature and motion sensitively and continuously. These multifaceted features indicated the great potential of the proposed patches for intelligent wound healing management.

## 2 Results and Discussion

### 2.1 Preparation of the Mucus, PAAm‐DN, and PNIPAm‐DN Hydrogels

**Figure 1:**
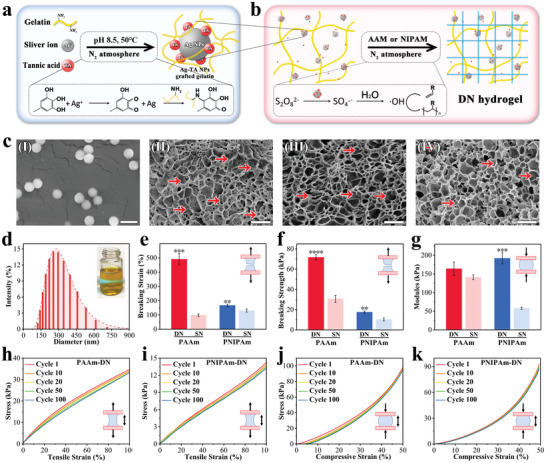
a) Schematic diagram of mucus synthesis. b) Scheme of the PAAm‐DN and PNIPAm‐DN synthesis. c) SEM images of (I) particles, (II) mucus, (III) PAAm‐DN, and (IV) PNIPAm‐DN. The scale bar is 500 nm in (I) and 2 µm in (II‐IV). d) Particle size distribution of Ag‐TA nanoparticles and the Tyndall effect of the nanoparticle dispersion. e–g) The tensile and compressive tests of hydrogels. e) Breaking strain. f) Breaking strength. g) Compressive modulus. The cyclic tensile test of h) PAAm‐DN and i) PNIPAm‐DN. j,k) The cyclic compressive test of j) PAAm‐DN and k) PNIPAm‐DN. For e–g), data are shown as mean ± SD, ^*^ *p* < 0.05, ^**^ *p* < 0.01, ^***^ *p* < 0.001, and *n* = 3.

In a typical experiment, different hydrogel components of the patch were fabricated in two‐step chemical reaction respectively, as shown in **Figure** **1**a,b. First, to obtain the Ag‐tannin (TA) nanoparticles grafted gelatin as snail‐inspired mucus, we used tannin to graft onto gelatin and reduce Ag^+^ into Ag‐TA nanoparticles in warm alkaline environment with vigorous stirring and nitrogen atmosphere, and thus the resulting mucus was a mixture of Ag‐TA nanoparticles and grafted gelatin (Figure 1a). Further, we mixed the mucus with a prepolymer solution of PAAm and PNIPAm with appropriate initiators to form double network (DN) hydrogel, namely PAAm‐DN and PNIPAm‐DN hydrogels, respectively (Figure 1b). The PAAm‐DN hydrogel constructed the tensile backing layer, while the PNIPAm‐DN hydrogel constructed the micro suction‐cup actuator array respectively (Figure S1a, Supporting Information). As expected, the infrared spectrum demonstrated the expected Michael addition reaction between gelatin and tannic acid as well as the free radical polymerization of PAAm and PNIPAm. The abundant hydroxyl groups and hydrogen bonding interactions in the resulting hydrogels were also indicated by FTIR, contributing to the excellent adhesion and mechanical properties (Figures S1b and S2, Supporting Information).

Given that the morphology and microstructure of both the Ag‐TA nanoparticles and the DN hydrogels are the prerequisites for excellent performance, their structural uniformity and constancy were studied thoroughly. Specifically, the scanning electron microscopy (SEM) images revealed the Ag‐TA nanoparticles with a uniform size (Figure 1c). Dynamic Light Scattering (DLS) further confirmed that the particle size was typically conformed to logarithmic normal distribution (R^2^ = 0.99682), concentrated ≈275 nm (Figure 1d). Tyndall effect also occurred under laser irradiation, indicating the uniform dispersity and stability of the Ag‐TA nanoparticles (Figure S3a,b, Supporting Information). It is because of the Coulomb repulsion and the presence of polymers that prevented agglomeration of the nanoparticles. After incorporating these nanoparticles into the hydrogel, a uniform distribution of the nanoparticles within the porous structure of hydrogel networks could be observed, as indicated by the arrow signs (Figure 1c; Figure S3c, Supporting Information). It showed that two types of double network hydrogels (PAAm‐DN and PNIPAm‐DN) had a denser network structure compared to the single‐network mucus. These uniformities guarantee the reusability and robustness of subsequent important properties of the hydrogel.

Profiting from strategies of double network, nano‐toughening, and introducing non‐covalent interactions, PAAm‐DN and PNIPAm‐DN exhibited excellent mechanical properties (Figure S3d, Supporting Information). In the tensile breaking test, the DN hydrogels displayed higher breaking strain and breaking strength (Figure 1e,f), which mainly due to the non‐covalent cross‐linking of gelatin. Additionally, the Ag‐TA nanoparticles replaced the sacrifice of robust covalent cross‐linking network in PAAm and PNIPAm. In particular, the breaking strain of PAAm‐DN increased nearly four times and its breaking strength was double of pure PAAm, as shown in Figure S4 (Supporting Information). In the compressive test, the modulus of both types of DN hydrogels were larger than those of corresponding single network (SN) hydrogels to a certain extent (Figure 1g; Figure S5, Supporting Information), which was derived from the more abundant interactions in the network. To simulate the actual repetitive deformation, cyclic compressive and tensile tests were designed within 100 cycles for each sample (Figure 1h–k). For both DN hydrogels, fatigue only occurred in the initial tension cycles and no longer decreased significantly in subsequent cycles. Besides, there was no obvious fatigue after compression cycles. Taken together, the DN hydrogel components of patches have excellent mechanical properties, which would greatly benefit long‐term wound management.

### 2.2 Preparation and NIR‐Responsive Behaviors of the Heterogeneous Patches

**Figure 2:**
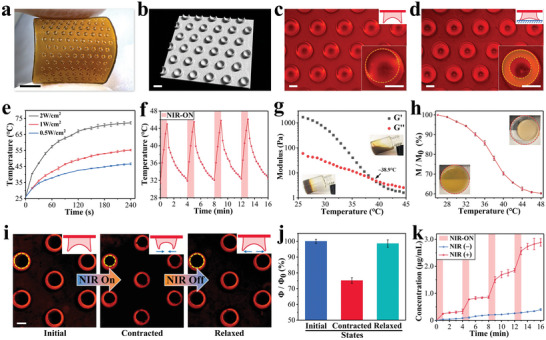
a) Digital photograph of the patch. The scale bar is 0.5 cm. b) 3D reconstruction of the patch via Micro‐CT. The scale bar is 1 mm. c) Optical micrograph of the initial micro suction‐cup array. Scale bars are 500 µm. d) Optical micrograph of suction‐cups gripped on the interface. The lower right corner is a partial magnified view. Scale bars are 500 µm. e) The photothermal response curves of the patch under different NIR power densities. f) Photothermal cycling curve of the patch. The red area means that the NIR is on during this period. g) The temperature‐moduli curve of mucus. G' and G”” are the storage modulus and loss modulus respectively. Inserted digital photographs refer to the gel and sol states of mucus. h) The shrinkage rate (*M*/*M*_0_)‐temperature curve of PNIPAm‐DN. *M*_0_ is the initial mass and *M* is the mass at a specific temperature. Digital photographs indicated the PNIPAm‐DN before and after contraction. i) The contract‐relax cycle of the suction‐cups. Scale bars are 500 µm. j) The contraction rate (*Φ*/*Φ*_0_) of the suction‐cup in different states, *Φ*_0_ is the initial diameter, and *Φ* is the diameter of a specific state. k) The controlled drug release curve of the patch with and without NIR control. The model drug FITC‐BSA was used. The red area means that NIR is on during this period. For (e), (h), (j) and (k), data are shown as mean ± SD, *n* = 3.

As mentioned above, the backing layer and micro suction‐cup array were composed of PAAm‐DN and PNIPAm‐DN respectively. The heterogeneous patches were simply prepared via a step‐by‐step template perfusion method (**Figure** **2**a; Figure S6, Supporting Information). The Micro‐CT and fluorescence images showed orderly, uniform, and regular bowl‐like suction‐cups of the patch in the absence of pre‐pressure (Figure 2b,c; Figure S6, Supporting Information). Similar to an actual octopus, under pre‐pressure induction, the suction‐cup deformed elastically after contacting the interface, and the opening diameter increased accordingly when the pre‐pressure increased (Figure S7a, Supporting Information). When the pre‐pressure was removed, the suction‐cups still gripped on the interface with negative pressure induced by resilience (Figure 2d). Furthermore, a liquid film was found at the contact interface between the suction‐cup and the substrate, mainly due to capillary force, which also might contribute to the maintenance of negative pressure (Figure S7b, Supporting Information).

The presence of Ag‐TA nanoparticles would impart the patch with excellent photothermal conversion performance. Under NIR, the whole patch's temperature increased uniformly, and its heating rate together with equilibrium temperature could be controlled by different NIR energy densities (Figure 2e; Figure S8, Supporting Information). In detail, the energy density of 1 W cm^−2^ led to a relatively fast but mild heating profile, which was thus selected for all subsequent photothermal experiments. The patch could also generate cyclic photothermal behavior by temporally modulating the NIR with a periodic pulse signal (Figure 2f). Specifically, it completed four cycles from warming up to ≈45 °C to cooling down over 16 min. In addition, to flexibly and stably control the desired photothermal behavior of the patch, customized programmed NIR devices were constructed, as shown in Figure S9 (Supporting Information). These features demonstrated the reusability, robustness, and modulability of the photothermal behavior of the generated patch, which would benefit the accurate NIR control in the following wound management.

During the temperature‐changing cycles, the mucus of the patch showed a reversible gel‐sol transition behavior, which was derived from the temperature‐sensitive gelatin component (Figure 2g; Figure S10a, Supporting Information). The rheological test reflected that the storage and loss modulus of mucus decreased alongside the increase of temperature, and intersected at gelation temperature (Figure 2g). Specifically, the gelation temperature of the mucus was designed ≈38.9 °C to ensure a gel state at physiological temperature, while it could convert to a sol state under controllable NIR irradiation. Different from the gelation temperature (≈33 °C) of pure gelatin hydrogel at the same mass fraction, the increased gelation temperature of the mucus could be ascribed to the richer intermolecular interactions provided by tannic acid, etc (Figure S10b, Supporting Information). Meanwhile, the micro suction‐cup will shrink by volume phase transition (VPT) (Figure 2h; Figure S10c, Supporting Information). Specifically, the considerable shrinkage rate of the PNIPAm‐DN reached an equilibrium (≈60%) at elevated temperature. This should be owing to the dominance of hydrophobic force during temperature rise. When the NIR was off, the suction‐cup could recover. By analyzing the diameter change, we found that after a contraction of 25%, the suction‐cup could return to ≈100% of its original diameter, showing a responsive contract‐recover cycle under NIR control (Figure 2i,j). Benefiting from these, controlled drug release could be achieved using programmed NIR irradiation (Figure 2k). The step‐like‐shaped release profile demonstrated that the drug released slowly when the mucus was in the gel state and the suction‐cup relaxed without NIR irradiation. By contrast, the release rate was greatly enhanced by the solation of mucus and the contraction of the suction‐cup under NIR irradiation. This ensures unique NIR‐controllable precise drug administration according to different wound conditions.

### 2.3 NIR‐Controlled Adhesion Ability

**Figure 3:**
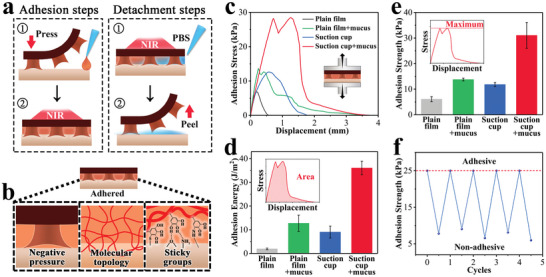
a) Schematic diagram of the controlled adhesion and detachment cycle of the patch. The whole process is mainly controlled by NIR. b) Schematic diagram of the multifaceted adhesive forces. c) The stress‐displacement curve of the patch. d) Adhesive energy of the patch. The schematic diagram shows that it is the integral area. e) Adhesive strength of the patch. The schematic diagram indicates that it is the maximum stress during separation. f) Cyclic adhesion and detachment experiments of the patch. For (d) and (e), data are shown as mean ± SD, *n* = 3.

Due to the temperature‐responsive properties, the adhesion and removal of the patch could also be controlled by the NIR laser (**Figure** **3**a; Figure S11, Supporting Information). The two steps for adhesion were simple, i.e., by pressing the patch on the mucus and then irradiating it with NIR. Under NIR, the suction‐cup contracted, and the mucus diffused and fullfilled the gap at the interface. The added mucus was then gelated and formed molecular topology when the NIR was off. This unique multifaceted adhesive strategy, namely suction‐cup+mucus has better universality (Figure S12a, Supporting Information). It involved the negative pressure of the suction‐cup, the molecular topology of mucus molecular chains, the intermolecular forces like hydrogen bonding of catechol groups in tannic acid and phase separation (Figure 3b; Figure S12b, Supporting Information). The removal of the patch also only takes two simple steps, namely solating the mucus of gel with NIR again and washing it away with cold PBS. In these processes, with the decreased temperature, the suction‐cup reabsorbed water and expanded. In addition, water molecules destroyed the interactions such as the hydrogen bonds of sticky groups. These effects make the patch non‐adhesive.

To demonstrate the respective and synergistic adhesion ability of the suction‐cup+mucus strategy in detail, mechanical tests were carried out on four groups, namely plane film, plane film+mucus, suction‐cup, and suction‐cup+mucus, respectively (Figure 3c). In the tests, the samples adhered to the pigskin were stretched vertically until separation to obtain the stress‐displacement curve. It demonstrated that the separation behavior of plane film was relatively the simplest with the least stress and displacement. Comparatively, the stress was enhanced in the plane film+mucus group because of the extra molecular topology and sticky groups of mucus, and displacement also increased, indicating the persistence coming from the mucus deformation (Figure S12c, Supporting Information). When the suction‐cup was present, the stress increased due to the additional negative pressure resulting from the cup. In this group, the elastic deformation of the suction‐cup structure also led to a similarly increased displacement. As hypothesized, both the stress and displacement were remarkably elevated owing to the synergetic effect of the adhesion factors derived from the mucus and suction‐cups in the suction‐cup+mucus group.

Furthermore, the adhesion strength and energy were quantitatively analyzed during the stretching test (Figure 3d,e). It also showed that the existence of mucus or the suction‐cup would benefit the adhesion. Particularly, the adhesion energy in the plane film+mucus group was ≈19 times that of the plane film group. Moreover, the combination of the two adhesion strategies brings about considerable adhesion strength (over 30 kPa). It is worth noting that the adhesion of the patch could be achieved repeatably (Figure 3f). In these tests, a tensile stress of 25 kPa was applied to verify the adhesion state of the patch. The adhesion strengths were then tested and recorded in five cycles of adhesion and the non‐adhesion states. The results indicated that within five cycles, the adhesion strength in non‐adhesive state was always significantly smaller than that of the adhesive state. A representative displacement‐stress curve of the non‐adhesive patch was also shown in Figure S13a (Supporting Information).

It is also worth mentioning that the patch can still maintain firm adhesion under relatively high temperatures, and simple NIR irradiation or temperature rise will not cause it to fall off. To prove this, qualitative and quantitative experiments were both carried out (Figure S13b,c, Supporting Information). In the qualitative experiment, we attached the patch to pig skin and placed it vertically. We used NIR irradiation and kept the temperature at 45 °C for ten minutes. During this period, there was no sign of patch detachment. In practical use, the patch rarely reaches this temperature, demonstrating its reliable adhesion at relatively high temperatures. In addition, quantitative adhesion experiments were performed, showing that the patch's adhesion strength was ≈14.5 kPa and 19.8 kPa at 42 and 45 °C, respectively. We roughly estimated that this was enough to withstand >500 times the weight of the patch itself, proving that it would not fall off by itself.

### 2.4 Multiple Physiological Monitoring Functions

**Figure 4:**
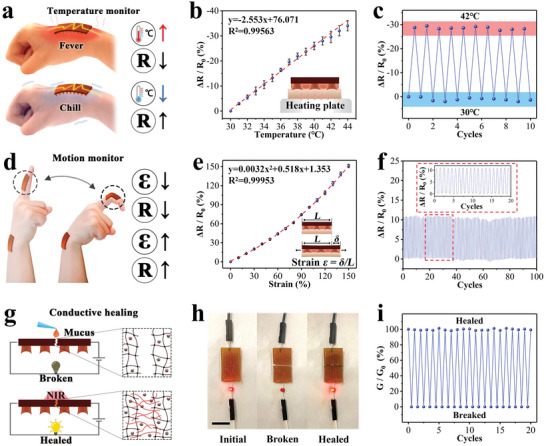
a) Continuous sensing of temperature. At different temperatures, the resistance changes differently. b) Resistance change rate (*ΔR*/*R_0_*)‐temperature curve of the patch. A linear function is used for fitting. c) The continuous monitoring of the temperature on the pigskin surface over 10 cycles. d) Continuous monitoring of motion by the patch. Under different strains, the resistance changes differently. e) Resistance change rate (*ΔR*/*R_0_*)‐strain curve of the patch. A quadratic function was used for fitting. Strain *ε* = *δ*/*L*, where the initial length is *L* and elongation is *δ*. f) Patch's monitoring of repeated motion over 100 cycles. Insert shows the detail of 20 cycles indicated. g) Schematic diagram of the conductive healing behavior, which is controlled by the NIR laser. h) Digital photographs of the patch conductive breaking‐healing process. The scale bar is 1 cm. i) The conductive breaking‐healing curve over 20 cycles. For (b) and (e), data are shown as mean ± SD, *n* = 3.

The good conductivity of TA‐Ag nanoparticles as well as the above‐mentioned structural stability and adhesion ability endowed the patch with flexible, continuous, and stable monitoring functionalities compared to traditional devices. Because the temperature is greatly correlated with the inflammation, infection, blood supply, etc., at the wound site, it is beneficial to monitor the temperature during wound healing management. In this system, the temperature could be monitored by studying the resistance change (**Figure** **4**a). To prove this, the patch was attached to the surface of the pigskin, and the change rate of resistance at a specific temperature was recorded. It demonstrated a negative temperature coefficient of resistance as −2.43% °C^−1^ throughout the body temperature range (30–44 °C), suggesting that the monitoring was sensitive (Figure 4b). The robustness of the online temperature sensing could also be proven based on the continuous and sensitive resistance change in cycles of temperature change on the pigskin (Figure 4c). In detail, its resistance change rate range uniformly undulated between 0 and 30% without noticeable shift or distortion within the ten cycles.

Continuous motion monitoring based on strain is of great significance for intractable motion wounds (such as wounds on joints, etc.) with frequent deformation (Figure 4d). Taking advantage of its ideal adhesion to the biological tissue, the patch experienced strain and a corresponding resistance change with the interface deformation. The strain response curve of the patch was recorded and calibrated in Figure 4e. It showed that the resistance change rate could reach 86% at 100% strain with a gauge factor of 0.85966, suggesting a good sensitivity and wide monitoring range of the patch. When attaching the patch to the finger and bending for 100 cycles, the uniform and periodic resistance change in ≈0–10% could be observed (Figure 4f; Figure S14a, Supporting Information). Notably, the motion monitoring was continuous, stable, and precise, and no adhesion failure or fracture occurred during the test.

Despite the satisfactory mechanical properties, motion sensors may still be broken by large strong external forces or sharp objects under extreme conditions, so their conductive healing ability is usually of great concern (Figure 4g). The conductive healing performance of the present patch was demonstrated by the indication of an LED light. After cutting off the patch, the LED light was off, suggesting the loss of conductivity; while the addition of mucus at the break site would heal the patch, and recover the conductivity to light up the LED again (Figure 4h). At the same time, the mechanical properties of the patch also recovered from ≈5 kPa to over 25 kPa for several cycles (Figure S14b,c, Supporting Information). These recoveries could be ascribed to the suture of the hydrogel network caused by the diffusion and topological adhesion of self‐cross‐linked mucus under NIR and the redispersion of Ag‐TA nanoparticles in the healed network (Figure 4g; Figure S14d,e, Supporting Information). In addition, the patch also showed its repeatable healing performance in 20 break‐healing cycles (Figure 4i). It was found that the conductivity could return to the original level after each healing even after complete cutdowns, revealing the effectiveness and reusability of the patch.

### 2.5 Biocompatibility and Dual Anti‐Microbial Activity

**Figure 5:**
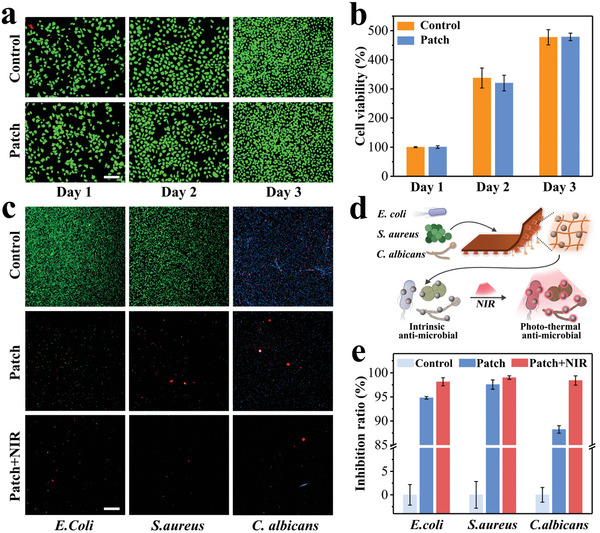
a) Live/dead staining images of HUVECs. b) Statistical analysis of CCK‐8 array. c) Live/dead staining images of *E. coli, S. aureus*, and *C. albicans*. d) Schematic diagram illustrating the dual antibacterial mechanisms of the patch, which can be controlled by NIR irradiation. e) Quantification of the inhibition rates of the *E. coli, S. aureus*, and *C. albicans* treated with the patch with or without NIR irradiation. All scale bars are 200 µm. For (b) and (e), data are shown as mean ± SD, *n* = 6.

For infected wound repair, patches are supposed to have excellent biocompatibility and efficacy for anti‐pathogen microbes at the same time. In this system, the biocompatibility of the patch benefitted from recognized biocompatible components including tannic acid, Ag, and gelatin‐based hydrogel matrix. For confirming such biocompatibility, human umbilical vein endothelial cells (HUVECs) were cocultured in the leachate derived from patches for three days. The live/dead staining of HUVECs demonstrated that most cells were alive in the patch group and the control group (**Figure** **5**a). Additionally, cell‐counting kit‐8 (CCK‐8) assays revealed that the cell viability of the patch group exhibited no statistical difference compared with the control group, and the increasing value over three days further confirmed good cell proliferation in both groups (Figure 5b). Further, co‐culture experiments for cytocompatibility were performed, as shown in Figure S15 (Supporting Information). The quantitative cell viability results showed that the cells co‐cultured with the material grew well and proliferated continuously within three days, and there was no significant difference compared with the control group. The live/dead staining showed good cell status with regular morphology and uniform size, and no obvious apoptosis or necrosis was observed. These two assays indicated the good biocompatibility of the patch.

In terms of anti‐bacteria activity, the patch could show broad‐spectrum antimicrobial capabilities, benefiting from both the intrinsic antimicrobial Ag together with tannic acid and the photothermal effect of Ag‐TA nanoparticles. By interacting with Gram‐negative/positive bacteria and fungi, treating with NIR or not, the antimicrobial ability of the patch was deeply investigated. Fluorescent staining of *E. coli* (gram‐negative bacteria), *S. aureus* (gram‐positive bacteria), and *C. albicans* (fungi) could directly and qualitatively illustrate the antimicrobial capabilities (Figure 5c,d). It showed that in the patch group, the population decreased significantly and a certain amount of death occurred compared to the control group showing viable and dense populations of microorganisms. Furthermore, the additional NIR irradiation would amplify the decline and the death trend. Meanwhile, the inhibition rate was calculated by the *OD*_600_ value (Figure 5e). Specifically, the inhibition rate exceeded 90% for both bacteria and 85% for fungi, and the treatment of NIR also elevated all the inhibition rates to over 98%.

### 2.6 In Vivo Wound Healing Effect

**Figure 6:**
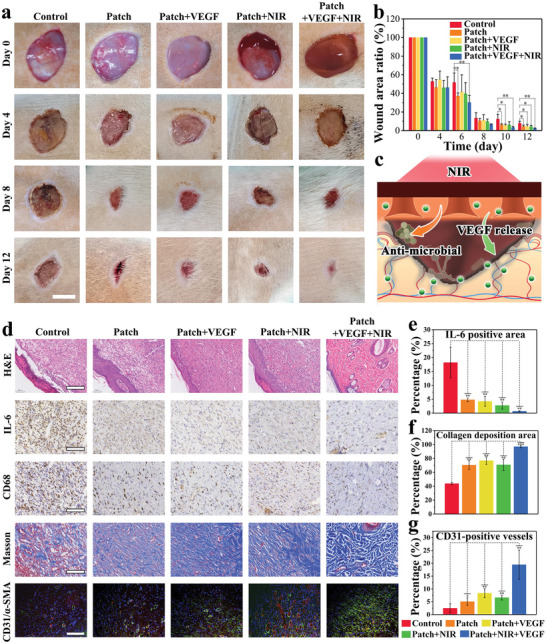
a) Digital photographs of the wounds in control, patch, and patch+VEGF, patch+NIR and Patch+VEGF+NIR groups. The scale bar is 1 cm. b) Quantification of the wound area ratio. c) Scheme of the synergistic effect of patch for wound healing. d) H&E and immunohistochemistry (IL‐6 and CD68), Masson, and immunofluorescence (CD31/*α*‐SMA) staining images. The scale bar for H&E is 200 µm, and others are 100 µm. e–g) Quantitative analysis of IL‐6, collagen deposition, and blood vessel density (CD31 positive area). For (b–d), one‐way ANOVA followed by Bonferroni's multiple comparison test. For (b) and (e–g), data are shown as mean ± SD, ^*^ *p* < 0.05, ^**^ *p* < 0.01, ^***^ *p* < 0.001, and *n* = 3.

Encouraged by the aforementioned features, we further explored the in vivo wound healing effect of the drug‐loaded patch using an infected cutaneous full‐thickness defect model in rats. The wounds were treated with PBS (control group), patch, patch+VEGF, patch+NIR, and patch+VEGF+NIR, respectively. The wound healing process was photographed and recorded on days 0, 4, 6, 8, and 12 (**Figure** **6**a). As compared to the control group, all patch‐treated groups displayed a more significantly reduced lesion area over time, and some positive performance such as hair coverage could be observed. Quantitative analysis confirmed a significantly higher healing rate in the treated groups than that in the control group on day 12 (Figure 6b). VEGF is known to stimulate wound healing through multiple mechanisms including epithelialization, collagen deposition, and angiogenesis, and the NIR irradiation could play an important role in the anti‐microbial activity and drug release (Figure 6c). Therefore, the patch+VEGF+NIR group possessed the best wound closure rate among all groups, showing significant differences with all the other treatment groups (p < 0.05) at day 12, suggesting the synergistic wound healing effect of the patch, NIR, and VEGF.

To further assess the wound beds and epithelization condition, hematoxylin, and eosin (H&E) staining was conducted on day 12. As shown in Figure 6d, in the control group, areas without granulation tissue formation still existed, an inflammatory response was observed in the surface epithelium. In patch+VEGF and patch+NIR groups, the regenerated epithelial layer integrated better with the surrounding normal tissues than that in the patch group. Furthermore, in the patch+VEGF+NIR group, a normal morphology of arranged epidermal and dermal layers appeared in the lesion site, confirming the synergistic effectiveness of VEGF and NIR stimulation. We next conducted immunohistochemistry analysis on the proinflammatory cytokine interleukin‐6 (IL‐6) and CD68 (Figure 6d). Statistical analysis indicated less inflammation in all treated groups, especially in patch+VEGF+NIR group (Figure 6e). Apart from the anti‐infection, attenuated inflammation, and multi‐promotive tissue repair as aforementioned, these excellent results could also be attributed from the inherent ROS scavenging activity of the Ag‐TA nanoparticles (Figure S16, Supporting Information).

Given that collagen can support the functional cells for wound contraction healing, we further investigated the effect of the patch on collagen deposition during wound healing. Masson's trichrome staining was conducted and the results showed the collagen deposition was accelerated in all treated groups as compared to that in the control group (Figure 6d,f). Among all groups, patch+VEGF+NIR group had the best performance, probably because of the synergistic effects as mentioned before. Additionally, the gelatin component in the patch may also promote collagen synthesis and deposition. Angiogenesis is known as a crucial factor to indicate the damaged blood vessel repair during wound healing. Thus, immunofluorescence staining of CD31 (red) and *α*‐SMA (green) was performed to indicate vascular endothelial cells of new vessels and vascular smooth muscle cells of mature blood vessels, respectively (Figure 6d). For the control group, there were few positive areas for CD31 and *α*‐SMA. Furthermore, compared to others, higher expressions of CD31 and *α*‐SMA were observed in the patch+VEGF and patch+VEGF+NIR groups (Figure 6e). Notably, the patch+VEGF+NIR group showed the highest angiogenic level among all groups, which further confirmed the synergistic wound healing efficacy of the patch, NIR, and VEGF.

## 3 Conclusion

In summary, inspired by snail mucus and octopus suction‐cup, we designed a highly integrated hydrogel patch with NIR‐controlled adhesion, anti‐microbial, drug release, and physiological monitoring features for wound healing management. The patch was composed of mucus, a DN hydrogel micro suction‐cup array, and a tensile backing layer, which could be derived from simple preparation manners. The photothermal effect of the Ag‐tannin nanoparticles and the gel‐sol transition of gelatin endowed the mucus with dual anti‐microbial as well as temperature‐sensitive activities. In addition, the microcosmic stability of the patch ensured good mechanical properties as well as reproducible optical thermal effects, from which the loaded VEGF could be controllably released for wound healing. The resultant patch also showed its potential in sensitive and continuous wound physiology monitoring including temperate and motion sensing because of its robust flexibility, self‐healing ability, and conductivity. At the in vivo level, the patch also showed improved wound healing for its antimicrobial, epithelialization, collagen deposition, and angiogenesis performances. All of these features indicated the great practicability and innovation of the proposed patch in the field of intelligent wound healing management.

We believe that such simple preparation, integrated functions, good performance and precise controllability will be the development trend of medical patches. Although it is currently limited in skin wound management with NIR control, in the future, this patch is expected to become a more universal intelligent wound management platform for a wider range of situations. Such bio‐inspired medical patches can be loaded with more diverse drugs on demand and are expected to further integrate with the heating effect of magnetic field, ultrasound, and other control means with deeper tissue penetration for remote internal treatment. It also has the potential to be further integrated with wireless modules to realize remote monitoring of wounds. In conclusion, the present patch is expected to exert greater value as a general platform by further integrating with other advanced technologies for the foreseeable future.

## 4 Experimental Section

The Experimental Section is available in the Supporting Information.

## Conflict of Interest

The authors declare no conflict of interest.

## Author Contributions

Y.J.Z., L.R.S., and K.C. conceived the idea and designed the experiment. W.Z.L. and R.K.H. conducted experiments and data analysis. W.Z.L., Y.R.Y., and R.K.H. wrote the manuscript. Y.R.Y., X.C.W., and P.X.L participated in the scientific discussion.

## Supporting information

Supporting Information

## Data Availability

The data that support the findings of this study are available from the corresponding author upon reasonable request.
