# Supplementary material for: Multi‐Bioinspired Functional Conductive Hydrogel Patches for Wound Healing Management
Source: Adv Sci (Weinh). 2023 Jun 27;10(25):2301479. doi: 10.1002/advs.202301479 (PMC10477846; doi:10.1002/advs.202301479)
Supplement: Supplementary file 1 — Supporting Information [file ADVS-10-2301479-s001.pdf]

ADVANCED SCIENCE
Open Access

Supporting Information

for *Adv. Sci.*, DOI 10.1002/advs.202301479

Multi-Bioinspired Functional Conductive Hydrogel Patches for Wound Healing Management

Wenzhao Li, Yunru Yu, Rongkang Huang, Xiaocheng Wang, Puxiang Lai, Kai Chen, Luoran Shang* and Yuanjin Zhao**

Supporting Information

Article type: Research Article

Multi-bioinspired functional conductive hydrogel patches for wound healing management

Wenzhao Li, Yunru Yu, Rongkang Huang, Xiaocheng Wang, Puxiang Lai, Kai Chen, Luoran Shang*, Yuanjin Zhao**

Dr. W. Li, Dr. Y. Yu, Dr. X. Wang, Prof. Y. Zhao
Department of Rheumatology and Immunology, Nanjing Drum Tower Hospital, School of Biological Science and Medical Engineering, Southeast University, Nanjing 210096, China
E-mail: yjzhao@seu.edu.cn

Dr. W. Li, Dr. Y. Yu, Dr. X. Wang, Prof. L. Shang, Prof. Y. Zhao
Oujiang Laboratory (Zhejiang Lab for Regenerative Medicine, Vision and Brain Health), Wenzhou Institute, University of Chinese Academy of Sciences, Wenzhou, Zhejiang 325001, China

Prof. L. Shang
Shanghai Xuhui Central Hospital, Zhongshan-Xuhui Hospital, and the Shanghai Key Laboratory of Medical Epigenetics, the International Co-laboratory of Medical Epigenetics and Metabolism (Ministry of Science and Technology), Institutes of Biomedical Sciences, Fudan University, Shanghai, China
E-mail: luoranshang@fudan.edu.cn

Prof. K. Chen
Department of Orthopedics, Shanghai Changhai Hospital, Naval Medical University, Shanghai 200433, China
E-mail: ch_kai@163.com

Dr. R. Huang
Department of General Surgery and Provincial Key Laboratory of Colorectal and Pelvic Floor Diseases, Guangdong Institute of Gastroenterology, The Sixth Affiliated Hospital, Sun Yat-sen University, Guangdong 510655, China

Dr. W. Li, Prof. P. Lai
Department of Biomedical Engineering, The Hong Kong Polytechnic University, Hong Kong SAR, China

1. Experimental Section

Materials

Gelatin from porcine skin, acrylamide, N-Isopropylacrylamide, and fluorescent brightener 28 were purchased from Sigma Aldrich, USA. Tannic acid, AgNO3, NaOH, HCl, Bis-acrylamide, potassium peroxodisulfate, Rhodamine B and N,N,N',N'-Tetramethylethylenediamine were from Macklin, China. Bovine serum albumin (BSA) labeled by fluorescein isothiocyanate (FITC), tryptone soy broth, and liquid sabourand medium were bought from Solarbio, China. VEGF was obtained from PeproTech. *E. coli* (ATCC8739), *S. aureus* (ATCC9144), and *C. albicans* (ATCC10231) were obtained from Shanghai Bioresource Collection Center, China. HUVECs and endothelial cell medium were purchased from ScienCell, China. The CCK-8 and live/dead cell viability kit were derived from Thermo Fisher Scientific, USA. SYTOX Green and propidium iodide were achieved from Keygen Biotech, China. Ecoflex was purchased from Smooth-On, Inc., USA. Arduino board (UNO Rev3) was obtained from Allchips Ltd., China.

Preparation of the mucus

The mucus referred to the Ag-TA nanoparticles grafted gelatin. To obtain it, under the atmosphere of nitrogen protection and 50°C water bath heating, gelatin (200 mg mL^{-1}) and tannic acid (1 mg mL^{-1}) were dissolved in ultrapure water (10 mL). $AgNO_3$ (1 mL, 0.1 mol L^{-1}) solution was added under vigorous stirring. The pH was titrated by NaOH for maintaining at 8.5 during the 3 h reaction and finally adjusted to 7.4 with hydrochloric acid for termination.

Preparation of the SN hydrogels

To generate PAAm-SN and PNIPAm-SN, PAAm or PANPAm (200 mg mL^{-1}), potassium peroxodisulfate (5 mg mL^{-1}), and bis-acrylamide (2 mg mL^{-1}) were dissolved in ultrapure water as the prepolymer solution. During the crosslinking, N,N,N',N'-Tetramethylethylenediamine (1 μL mL^{-1}) was added into the prepolymer solution and then stood under nitrogen atmosphere until complete gelation.

Preparation of the DN hydrogels

Based on the protocols of SN hydrogels, ultrapure water was replaced with 50% (v/v) or 20% (v/v) mucus for PAAm-DN and PNIPAm-DN respectively. When crosslinking, N,N,N',N'-Tetramethylethylenediamine (0.5 μL mL^{-1}) was added into the prepolymer solution of PNIPAm-DN.

The prepolymer of PAAm-DN could self-crosslink without N,N,N',N'-Tetramethylethylenediamine due to the catalytic performance of Ag-TA nanoparticles. Both the two DN hydrogels stood under the nitrogen atmosphere until complete gelation.

Preparation of the heterogeneous patches

A step-by-step template perfusion method was used to obtain the heterogeneous patch. Firstly, the prepolymer solution of PNIPAm-DN was filled into the suction-cup array of the mold under the vacuum and the prepolymer solution of PAAm-DN was perfused again as the backing layer. After complete gelation, the patch was carefully rolled over. The backing layer was 2 mm. Mucus was added in an amount of 50 µL cm^{-2}. Unless otherwise mentioned, all patches contain added mucus.

Mechanics performance test

In compressive tests, the compression started from the original height to 50% strain. The modulus was the ratio of stress to strain at this time. In the tensile tests, the tension started from the original length to break. In the cyclic tests, strains of hydrogels were cycled between 0-50% for compression and 0-100% for tension.

Photothermal performance demonstration

The hydrogel patch of 2.0 cm × 2.0 cm was put under the NIR laser of 808 nm. The temperature was recorded. The NIR irradiation was modulated by an Arduino board. Specifically, in the photothermal cycling experiment, unless otherwise mentioned, the NIR was modulated to be 1 W cm^{-2} for 1 min and then turned off for 3 min as a cycle. The test was repeated for 4 cycles.

Contract-relax experiment of the suction-cups

Micro suction-cup arrays of the patch were labeled with Rhodamine B. The initial diameter was recorded as Φ_0. Then the patch was irradiated with NIR and kept at 45℃ The diameter (Φ) at this time was also recorded. Then NIR was turned off, and the diameter (Φ) was recorded again after the size of the suction-cups became stable. The contraction rate was equal to Φ/Φ_0. The diameter was measured by ImageJ.

Drug release experiment

The model drug FITC-BSA at a total amount of 50 μg was uniformly loaded into the mucus and suction-cups of the patch. The total mass of the patch was 1g. Then the patch was immersed in 5 mL PBS solution under 37℃. For NIR(+) groups, the same NIR irradiation model as the previous photothermal cycle was applied additionally. The solution was sampled and drug release was measured by multimode microplate reader.

Adhesion experiments

A patch of 2.0 cm × 2.0 cm was pre-pressed under a force of 4 N to the same size pigskin while 200 μL of sol mucus was added before the force was withdrawn under 37℃. Then the patch was exposed to the NIR irradiation for 1 min to maintain the temperature at 45 ℃. After the temperature returned to 37 ℃, tensile tests were performed. For removal, the patch was again exposed to the NIR irradiation for 1 min to recover the temperature to 45℃ and 10 mL of PBS at 25℃ was used to wash mucus away from the gap. For cyclic adhesion and detachment experiments, the test preparation in the adhesive state was the same as above and stopped when the stress reached 25 kPa. The non-adhesive state test was performed immediately after PBS washing.

Physiology indicator monitoring

For the temperature-resistance change rate ($\Delta R/R_0$) curve, the patch attached to the surface of the pigskin was heated and the resistance was recorded. For temperature cycle monitoring, the temperature of the hot stage was cycled between 42℃ and 30℃, and the resistance was recorded.

For the strain-resistance change rate ($\Delta R/R_0$) curve, in order to obtain more standard results, the patch was attached to the neatly shaped and highly elastic rectangular Ecoflex film surface, which was used to simulate the biological tissue. The resistance of the patch was then recorded during stretching. For repeated motion monitoring, a patch was attached to the phalangeal joint of the index finger, and resistance was continuously recorded. All the tests were carried out with a bench digital multimeter, and all the individual sampling points of resistances were read after stabilization. When serve as a non-invasive flexible electronic sensor applied on human skin, it does not require approval according to local laws.

***In Vitro* biocompatibility assay**

For leachate biocompatibility assay, the HUVECs of initial density equal to 10^4 cells ml^{-1} were cultured in three days. For the control group, the endothelial cell medium was used. For the patch group, the patch was soaked at a ratio of 0.2 g patch into 1 ml endothelial cell medium during 1 day to obtain leachate, which is referred to the ISO 10993-12 standard. Then filtered leachate was used for cell culture. For co-culture assay, the 3T3 cells of initial density equal to 10^4 cells ml^{-1} were cultured in three days. In the experimental group, extra 0.1 or 0.2 g patch was added to each 1 ml of culture medium and co-cultured with cells. The live/dead staining and CCK-8 assay were carried out strictly according to the kit instructions every day.

Dual anti-microbial experiment

E. coli, *S. aureus* and *C. albicans liquid* were prepared (OD_{600} = 0.5). For the control group, 50 µL bacterial/fungal liquid was inoculated in 2.5 mL of the corresponding liquid medium in a 6-well plate. For the patch group, 50 µL bacterial/fungal liquid was inoculated evenly on a 0.5 g patch and interacted for 15 minutes. For the patch+NIR group, additional NIR irradiation consistent with the previous photothermal cycle was applied. Then the patches were put into a 2.5 mL liquid medium (namely each 0.2 g patch add into 1 ml medium). Bacteria and fungi were cultured for 8 and 24 h respectively. Finally, OD_{600} test and fluorescent staining were performed on each group. The following equation is used for the calculation:

$$\text{Inhibition rate}=(1-OD_{experimental}/OD_{control})*100\%$$

Herein, $OD_{experiment}$ and $OD_{control}$ represent OD_{600} of experimental group and control group respectively. For fluorescent staining, live bacteria were stained green by SYTOX Green, fungi were stained blue by fluorescent brightener 28, and dead bacteria and fungi were stained red by propidium iodide.

***In Vivo* animal experiment**

Male Sprague-Dawley rats of 200-250 g (Sun Yat-sen University) were equivalently and randomly divided into 5 groups. The backs of rats were shaved, and pieces of full-thickness skin with 1.0 cm diameter on the back were excited to create a skin wound model, then *S. aureus* (20 µL, 10^9 CFU mL^{-1}) was added to the round wound bed. For the control group, the rats were only treated with PBS solution. For the patch group, a patch with a diameter of 1.2 cm adhered to the wound. For the

patch+VEGF group, additionally, 0.25 μg mL^{-1} VEGF was uniformly loaded into the mucus and suction-cups of the patch. For patch+NIR group, additional cycled NIR irradiation was applied. For the patch+VEGF+NIR group, the above-mentioned VEGF and NIR were administered simultaneously. The wound changes were photographed with measurement on days 0, 4, 6, 8, and 12, using ImageJ for statistics. All rats were sacrificed after 12 days. The tissues over the wound bed were harvested. Then it was treated with 4% paraformaldehyde, embedded in paraffin, and cross-sectioned. Subsequently, histological analyses including immunohistochemistry and immunofluorescence staining were tested by Wuhan Servicebio Technology Co., Ltd., China. The whole animal experiments were reviewed and approved by the Animal Ethics Committee of Guangzhou Huateng Biomedical Technology Co., Ltd, the approval number is HTSW220933.

Characterization

The microstructures for the nanoparticles and hydrogels were observed through a scanning electron microscope (SU8010, Japan). Zeta potential and size measurements were performed by Nanophox Zetasizer (ZEN3600). Optical and fluorescence microscopic images observed were gained by a stereomicroscope (Olympus BX51, Tokyo, Japan) and an inverted fluorescence microscope (ZEISS Axio Vert. A1, Germany). The micro-CT images were obtained by microcomputed tomography (SkyScan 1176, Bruker). The rheology characterization was tested by a rotational TA rheometer (DHR-2). The resistance was tested by a bench digital multimeter (DMM6500, Keithley, USA). The absorption spectrum of solution was measured by a multimode microplate reader (Varioskan LUX, ThermoFisher, USA). The mechanical tests were carried out with an electronic universal material testing machine (5944, Instron, USA). The 808 nm NIR was generated by a laser (DS3-808 nm -10 W BWT China). The temperature was recorded by an infrared thermometer (E5-XT, FLIR, USA).

Statistical analysis

Unless specifically mentioned, the whole data were expressed as mean ± standard deviations (SD). Statistical evaluation was analyzed using Student's t-test and the differences were considered statistically significant if $^{*}p < 0.05$, $^{**}p < 0.01$, $^{***}p < 0.001$ or $^{****}p < 0.0001$. Sample size (n) is detailed in the specific figure legends. All statistical analysis were conducted using SPSS software.

2. Supporting Figures

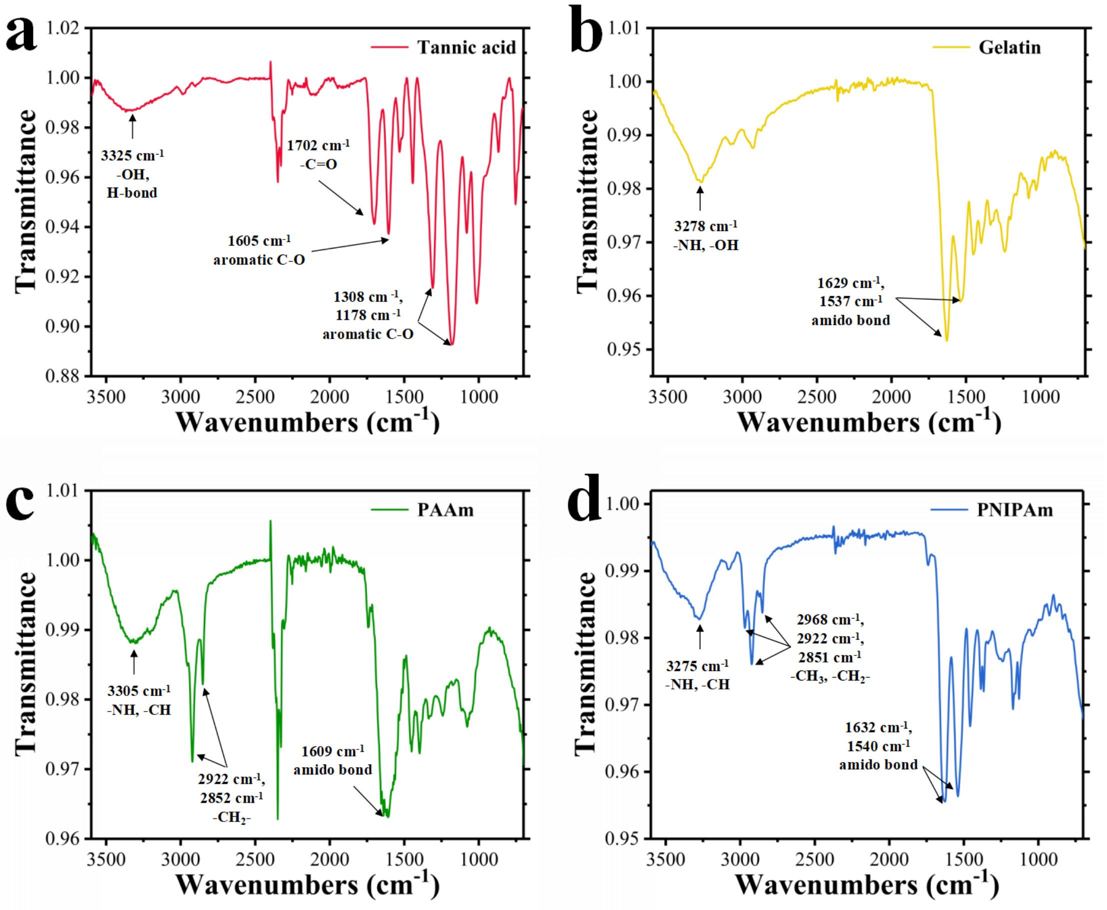

Figure S1. The FTIR spectra for the primary constituents of the patch. (a) Tannic acid. -OH stretch and H-bond were on 3325 cm^{-1}; C=O stretch was on 1702 cm^{-1}; C-O symmetrical was on 1605 cm^{-1}; C-O asymmetrical was on 1308/1178 cm^{-1}. (b) Gelatin. -NH and -OH stretch were superposed on 3278 cm^{-1}, amido bond (C=O, C-N and N-H) could be observed on 1629 cm^{-1} and 1537 cm^{-1}. (c) PAAm. Mainly, -NH and -CH stretch were superposed on 3305 cm^{-1}, $-CH_2-$ stretch was on 2922/2852 cm^{-1}, and amido bond was mainly observed on 1609 cm^{-1}. (d) PNIPAm. Mainly, -NH and -CH stretch were superposed on 3275 cm^{-1}. $-CH_2-$ and $-CH_3$ stretch was on 2968/2922/2851 cm^{-1}, and amido bond (C=O and N-H) was mainly observed on 1632/1540 cm^{-1}.

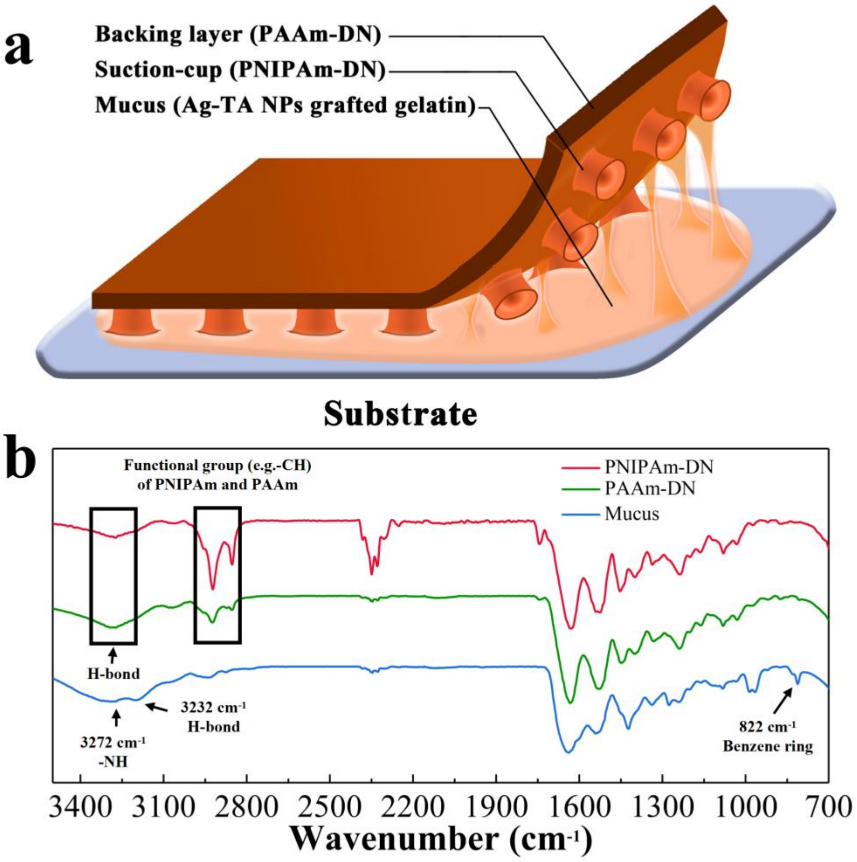

Figure S2. (a) Schematic diagram of the patch composition. The backing layer was constructed with the PAAm-DN hydrogel. The micro suction-cup actuator array was constructed with the PNIPAm-DN hydrogel. The mucus was composed of Ag-TA NPs grafted gelatin and filled the gaps in the interfaces. (b) The FTIR spectrum and characteristic peak of PAAm-DN, PNIAPm-DN, and mucus. For PNIAPm-DN and PAAm-DN, the superposed characteristic peak could be observed, indicating the co-existence of double network components. Compared with PAAm and PNIPAm of a single network, the peaks of PAAm-DN and PNIAPm-DN around 3300 cm^{-1} became broader due to hydrogen bond association. This reflected the interaction between networks. More specifically, for mucus, the -NH peak became weaker and the peak of tetrasubstituted\pentasubstituted benzene ring was on 822 cm^{-1}, indicating that the Michael addition occurred as expected.

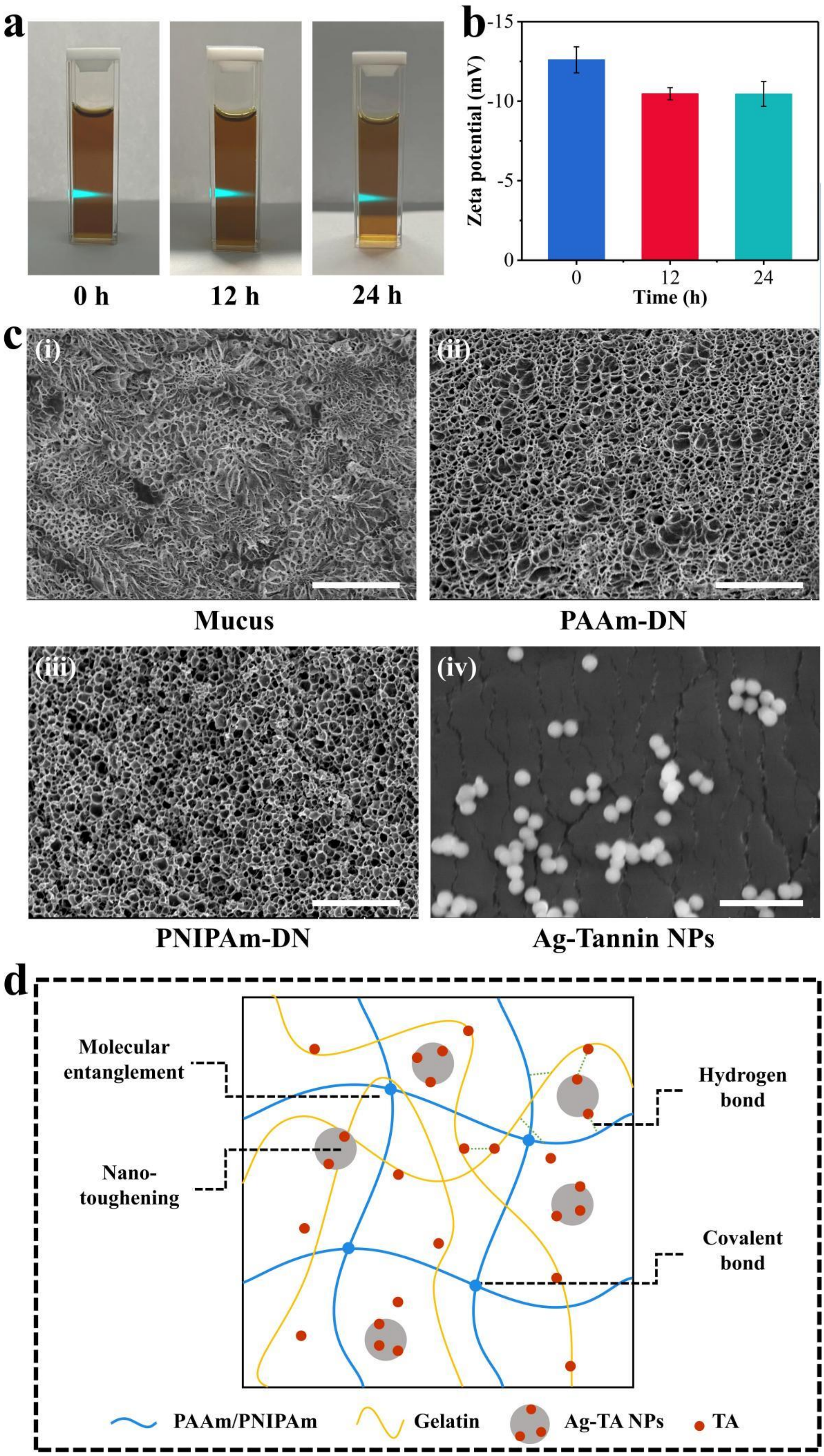
a
0 h
12 h
24 h
b
Zeta potential (mV)
-15
-10
-5
0
0
12
24
Time (h)
c
(i)
(ii)
Mucus
PAAm-DN
(iii)
(iv)
PNIPAm-DN
Ag-Tannin NPs
d
Molecular entanglement
Nano-toughening
Hydrogen bond
Covalent bond
PAAm/PNIPAm
Gelatin
Ag-TA NPs
TA

Figure S3. (a) Tyndall effect of the particles persisted after 24 hours stably, suggesting that the particles were well and stably dispersed in the solution. Scale bar is 1 cm. (b) Zeta potential of the particles was negative, suggesting that the Coulomb repulsion prevented agglomeration. In order to make the results observable and measurable, the mucus used in the experiment was diluted 5 times. (c) SEM images of (i) mucus, (ii) PAAm-DN, (iii) PNIPAm-DN and (iv) Ag-Tannin NPs at low magnification. The hydrogel network were all uniform and porous. Comparatively, the network of both DN hydrogels were relatively denser than the mucus. Scale bar are 10 μm for (i)-(iii) and is 1.5 μm for (iv). (d) Schematic illustration of the main interactions in the double network hydrogel. Rigid PAAm/PNIPAm were cross-linked by covalent bonds and non-covalently entangled with gelatin chains. Rich hydrogen bonds were generated mainly between tannic acid and molecular chains. The particles provided nano-toughening. In addition, there were coordination bonds between Ag and TA, π-π interactions between TA and other aromatic groups, etc. For (b), data are shown as mean $\pm$ SD, n = 3.

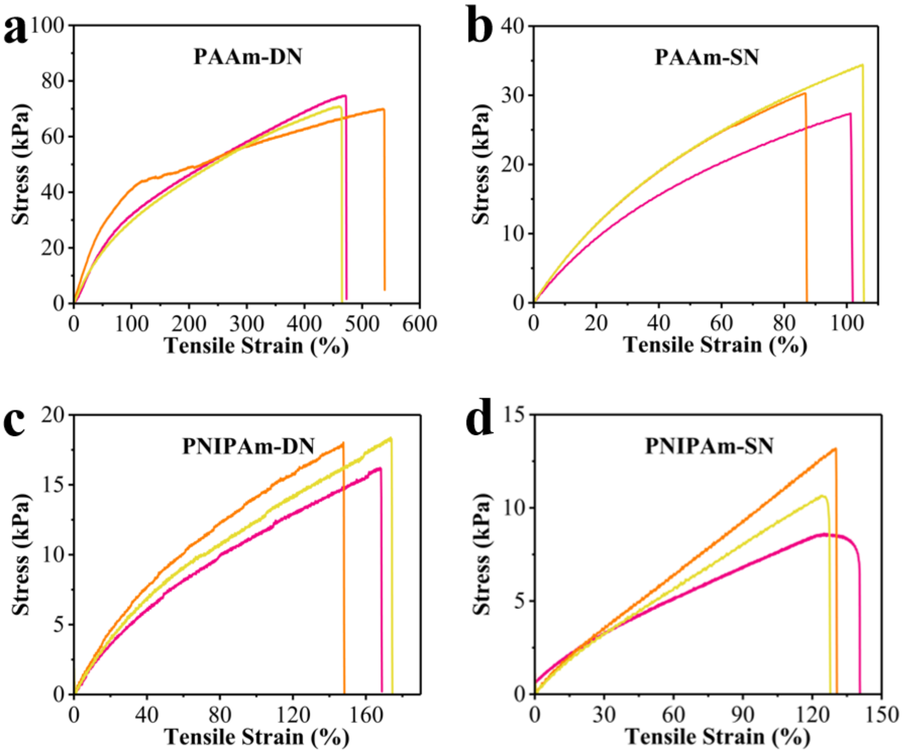

Figure S4. Detailed tensile strain-stress curves of (a) PAAm-DN, (b) PAAm-SN, (c) PNIPAm-DN, and (d) PNIPAm-SN. The hydrogel breaks at the highest point of the curve. The strain and stress at this time were recorded as breaking strain and strength, respectively.

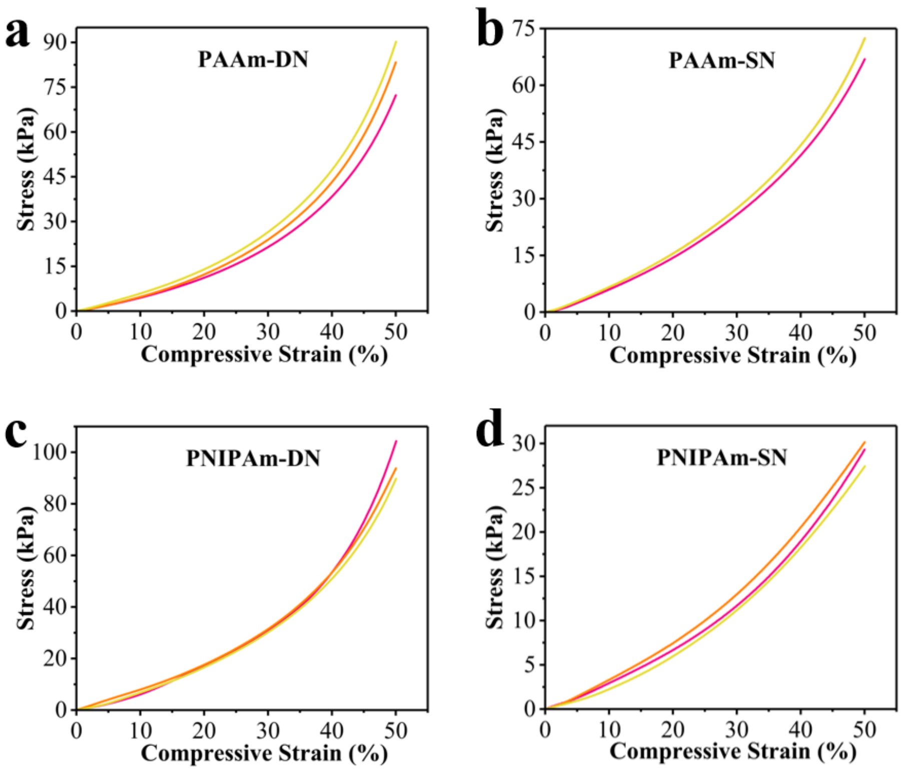

Figure S5. Detailed compressive strain-stress curves of (a) PAAm-DN, (b) PAAm-SN, (c) PNIPAm-DN, and (d) PNIPAm-SN. Compressive modulus is the ratio of stress to strain at end point of the curve.

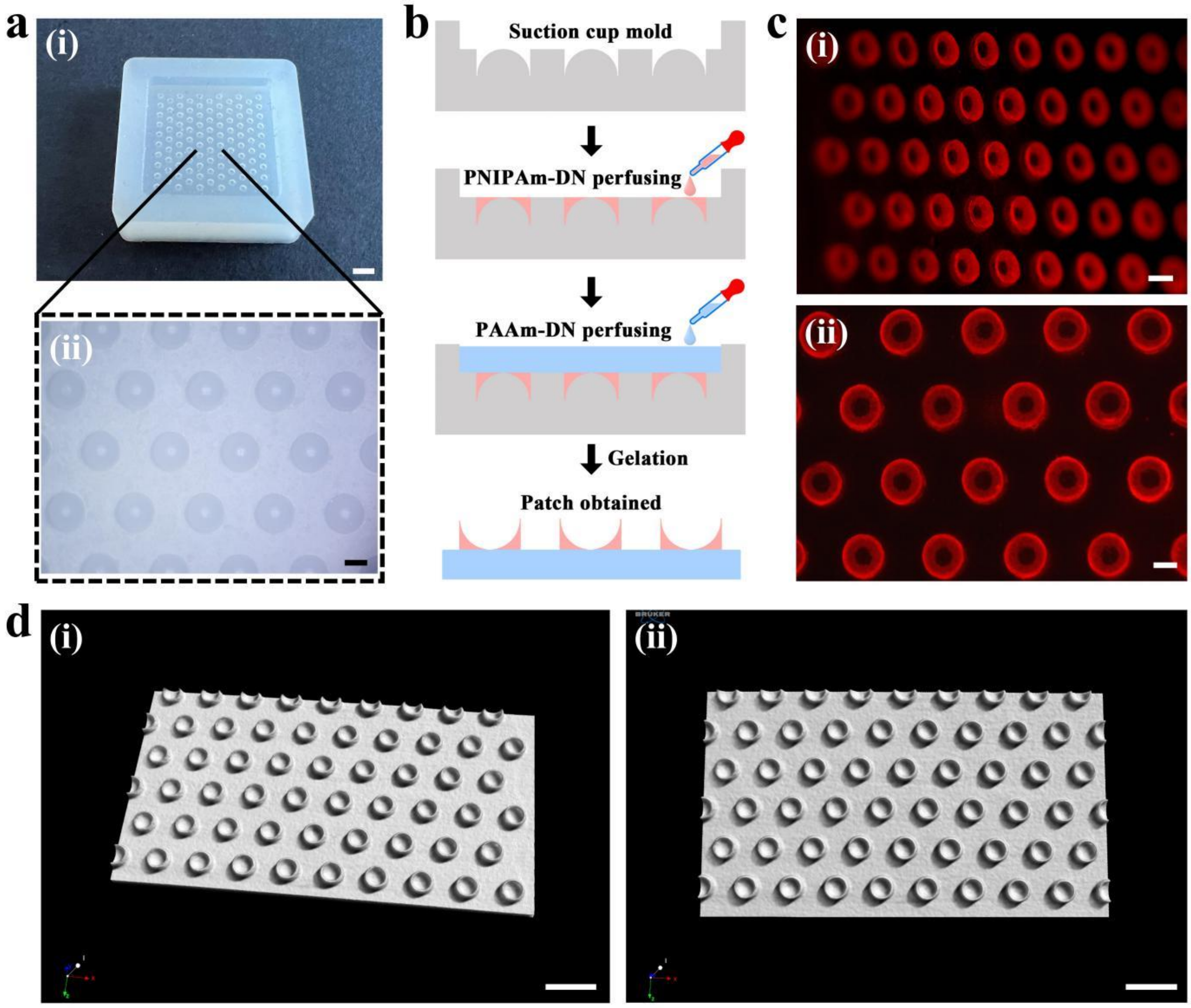

Figure S6. (a) Photograph and microscopic image of the suction-cup mold. Scale bars are 0.5 cm in (i) and 500 μm in (ii). (b) The step-by-step template perfusion method. The prepolymer solution of PNIPAm-DN and PAAm-DN was perfused sequentially. After gelation, the patch was carefully rolled over, and the mucus were added when applying. (c) Fluorescence images of the heterogeneous patches from the (i) oblique view and (ii) top view. The structure of the suction-cups were stained red. Scale bars are 1000 μm in (i) and 500 μm in (ii), respectively. (d) 3D reconstruction of the patch via Micro-CT in a larger field of view. The scale bars are 2 mm.

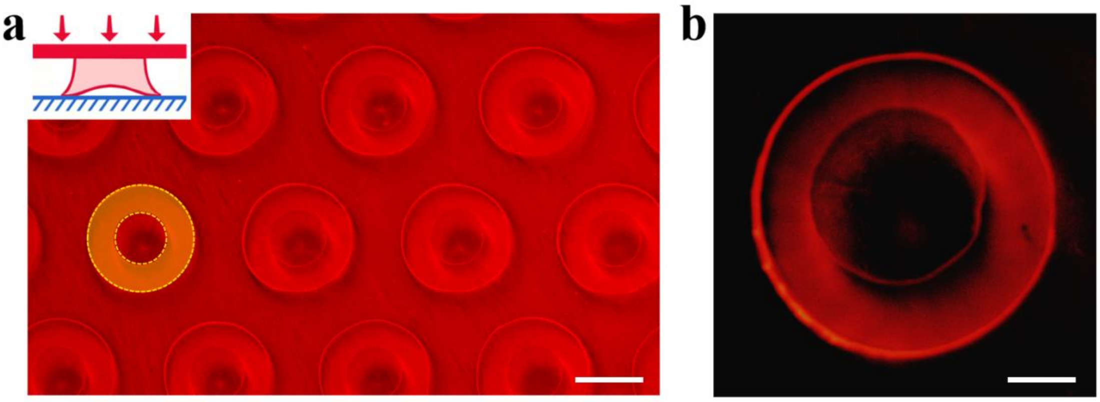

Figure S7. (a) Fluorescence image of suction-cups opening and contracted with the surface under pre-pressure. For demonstration, the patch was inverted and pre-pressed using a clear coverslip. The contact area was colored yellow. Scale bar is 500 µm. (b) A liquid film at the contact interface between the suction-cup and the substrate sealing the negative pressure. For demonstration, the patch was inverted, and a fluorescent solution was added on the interface for visualization of the liquid film.

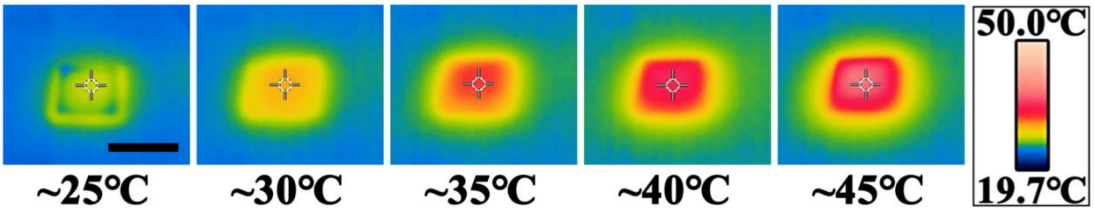

Figure S8. Thermal images of patches under NIR irradiation. The temperature of the patch increased from ~25°C to ~45°C with spatial uniformity. Scale bar is 2 cm.

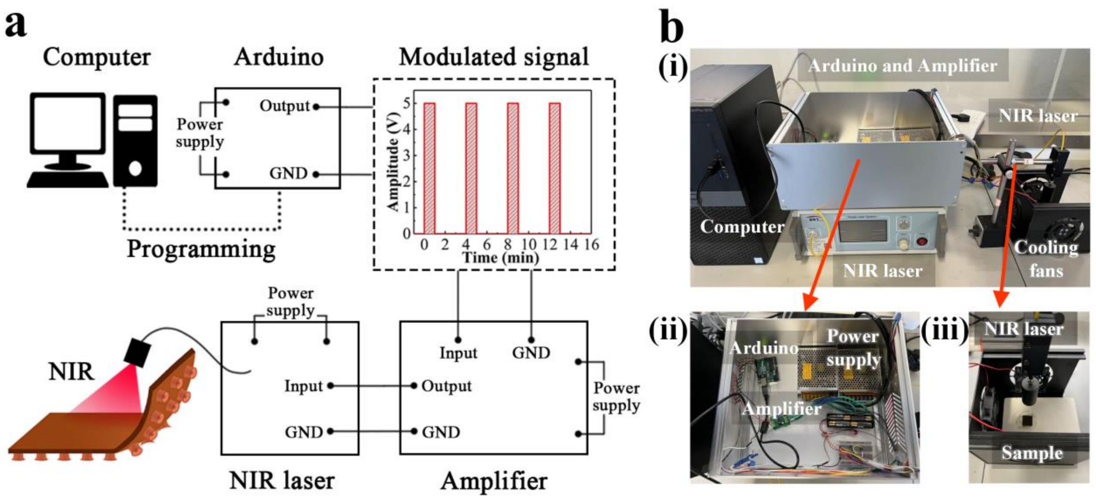

Figure S9. Customized programmed NIR devices. (a) The control signal of Arduino was programmed by the computer and output to the amplifier. Then the NIR laser was controlled to irradiate the patch sample. (b) Digital photographs of the setup.

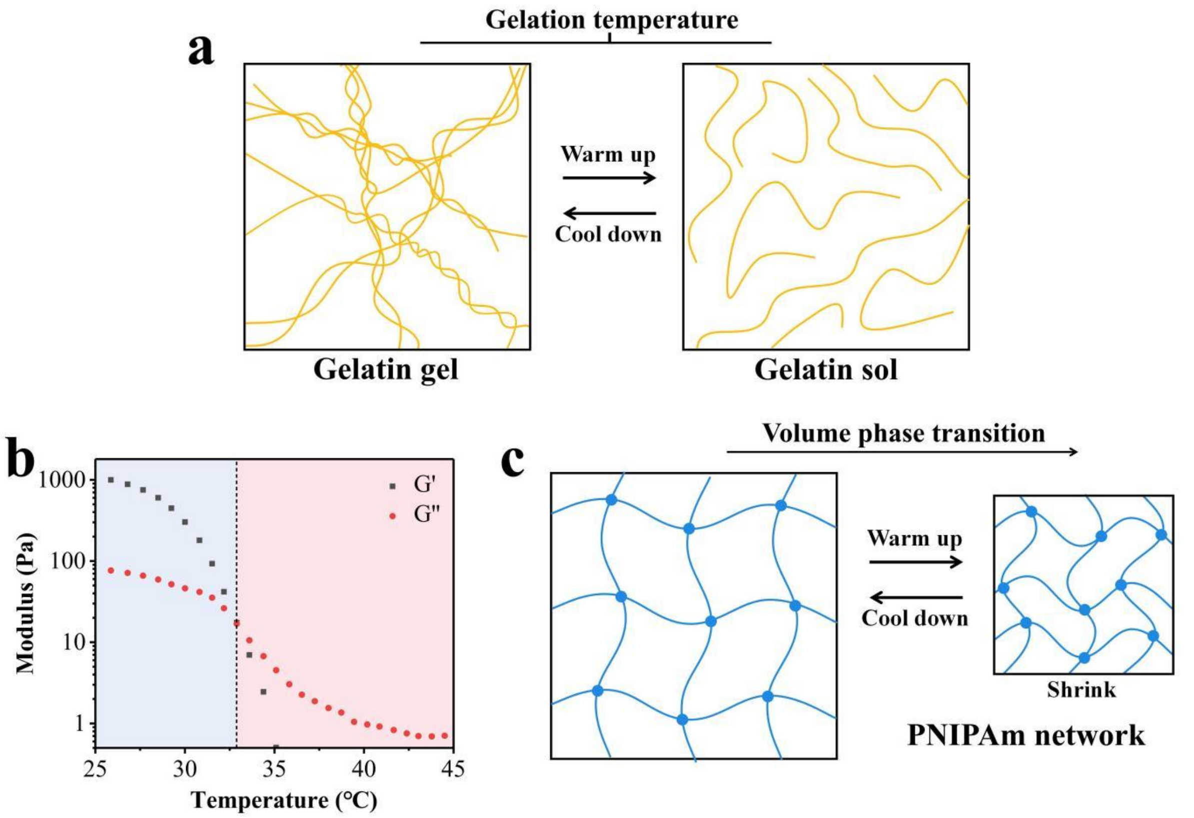

Figure S10. (a) Scheme of a reversible gel-sol conversion of the non-covalently cross-linked temperature-sensitive gelatin-based network. (b) The temperature-moduli curve of gelatin in the rheology test. G' and G'' represent the storage modulus and loss modulus. (c) Scheme of a volume phase transition (VPT) of PNIPAm. The PNIPAm network shrinks when warming up, whereas swells when cooling down, which is ascribed to its reversible change between the hydrophilic and hydrophobic states.

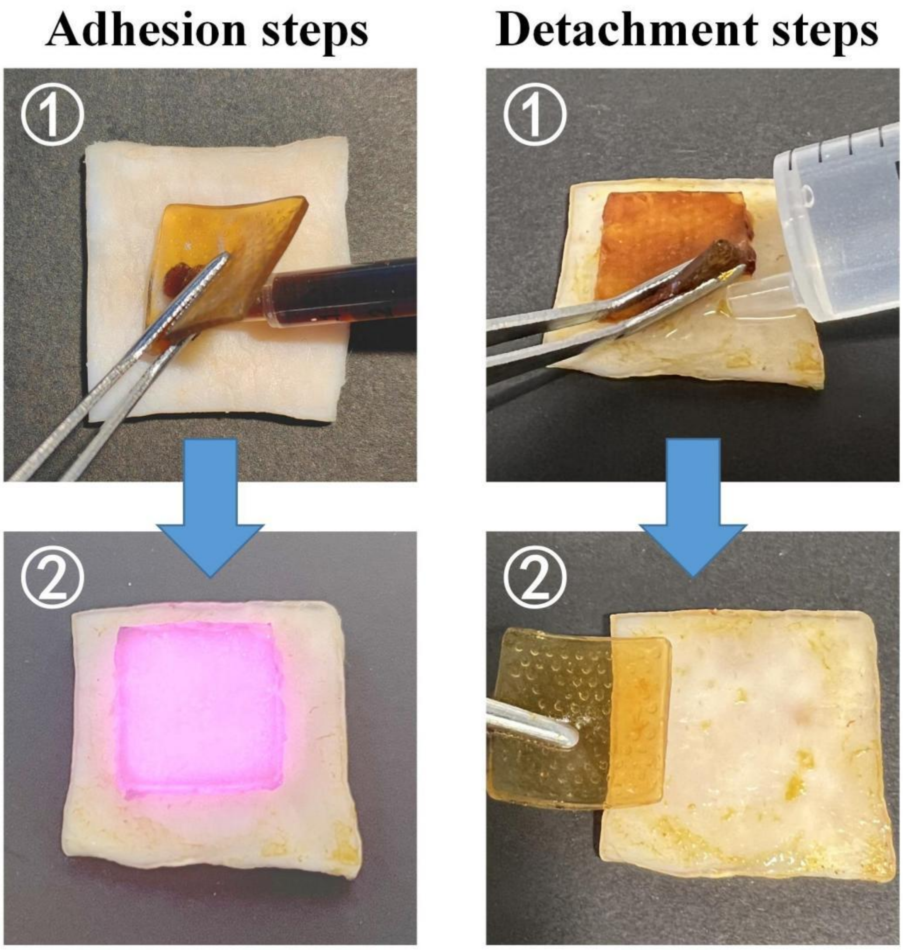

Figure S11. Digital photographs showing the controlled adhesion and detachment of the patch. Scale bar is 1 cm.

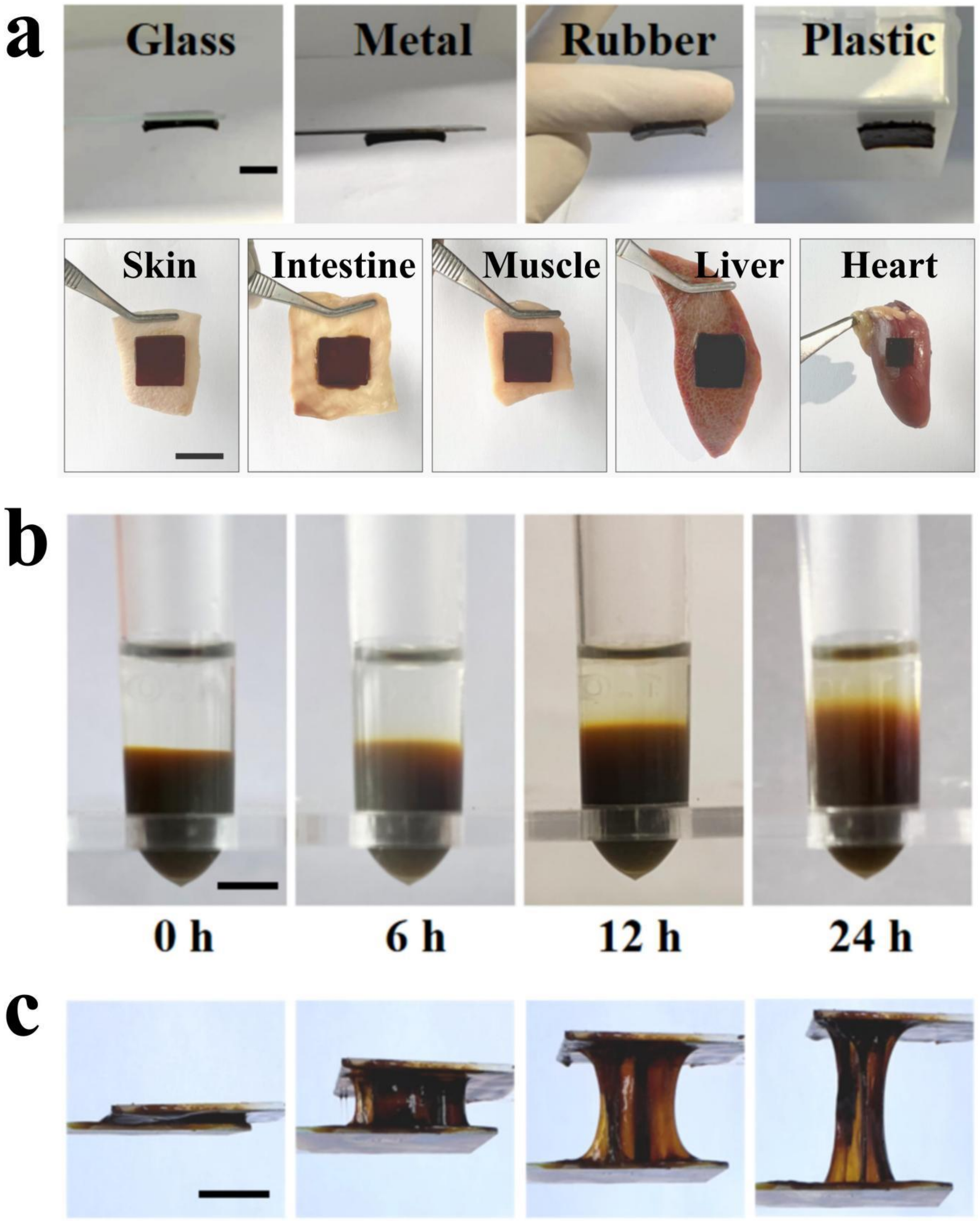

Figure S12. (a) The strategy named "suction-cup+mucus" mediates a wide range of adhesion capabilities, allowing the patch to adhere to a variety of surfaces. (b) At 37℃, mucus separated from water to a certain extent, ensuring the stability of underwater adhesion. (c) At 37℃, the mucus can deform to a large extent (λ>10), thereby dissipating energy. Scale bars are 1 cm in (a) and (c), and 0.5 cm in (b).

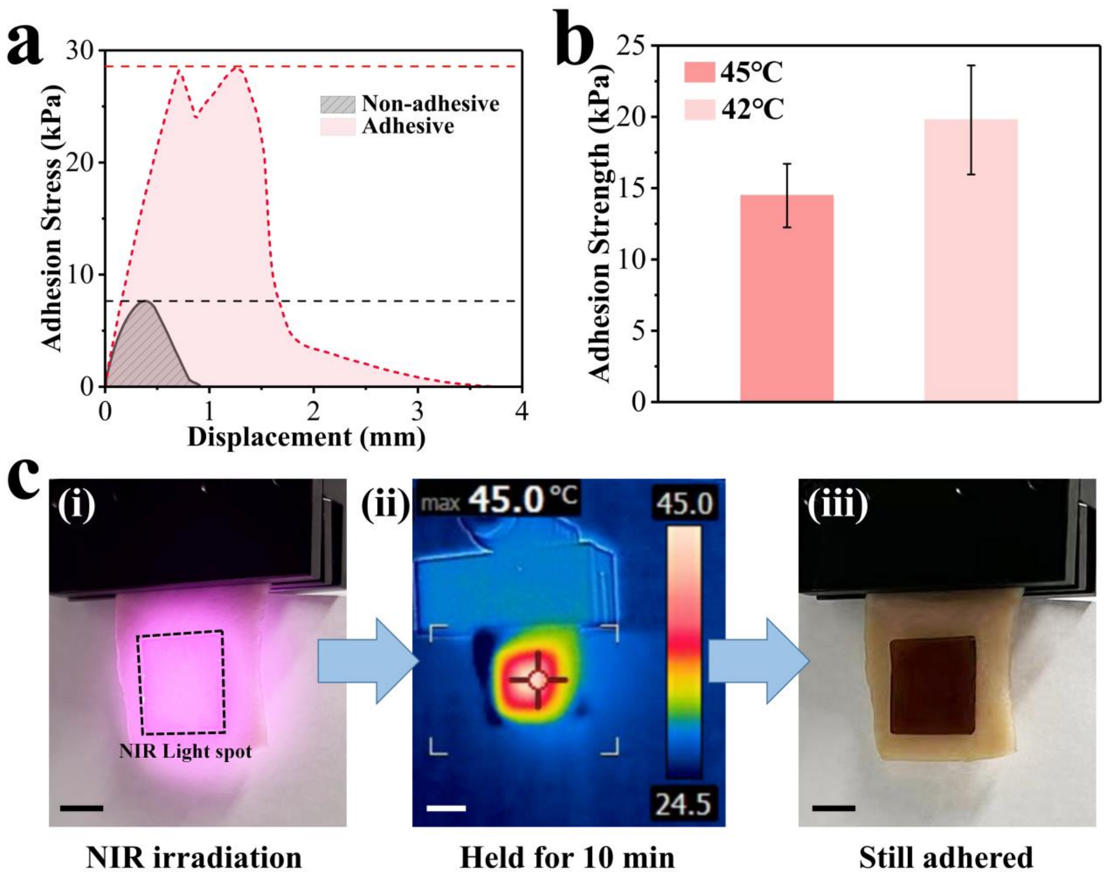

Figure S13. (a) Comparison of the displacement-stress curve between the non-adhesive and adhesive patches. The adhesive curve obviously showed a bigger area (adhesion energy) and height (adhesion strength) than the non-adhesive one, indicating the effectiveness of the strategy. (b) The adhesion strength at 42℃and 45℃ (c) The patch was attached to pig skin and placed vertically. NIR was used for irradiation and kept the temperature at 45°C for ten minutes. During this period, there was no sign of patch detachment. Scale bars are 1 cm. For (b), data are shown as mean ± SD, n = 3.

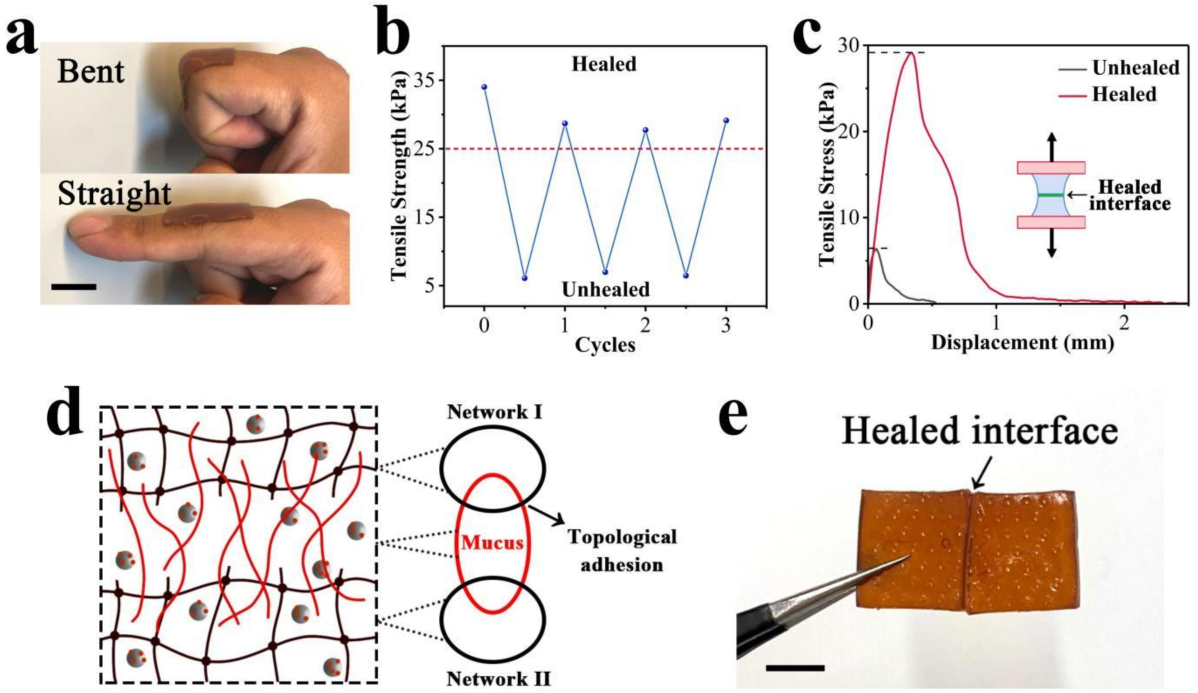

Figure S14. (a) The patch could firmly adhere to a finger joint regardless of the bent-straight motion. Scale bar is 1 cm. (b) The tensile strength in healed-unhealed cycles. (c) The stress-displacement curve of the healed and unhealed patch. (d) The schematic diagram of topological adhesion mediated controlled healing. (e) Photograph of the patch healed by mucus. Scale bar is 0.5 cm.

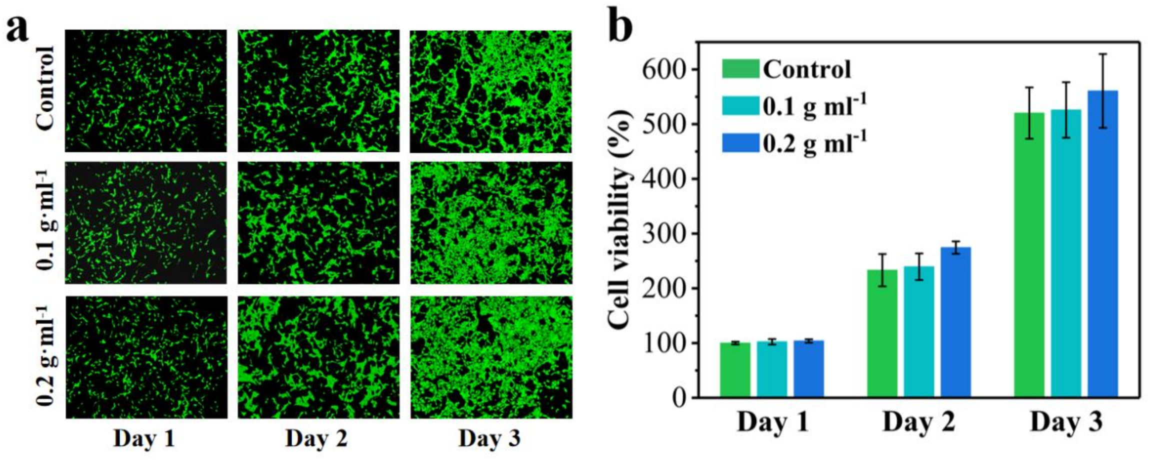

Figure S15. (a) Live/dead staining images of co-cultured cells. (b) Statistical analysis of CCK-8 array. In the experimental group, 0.1 or 0.2 g patch was added to each 1 ml of culture medium. For (b), data are shown as mean ± SD, n = 6.

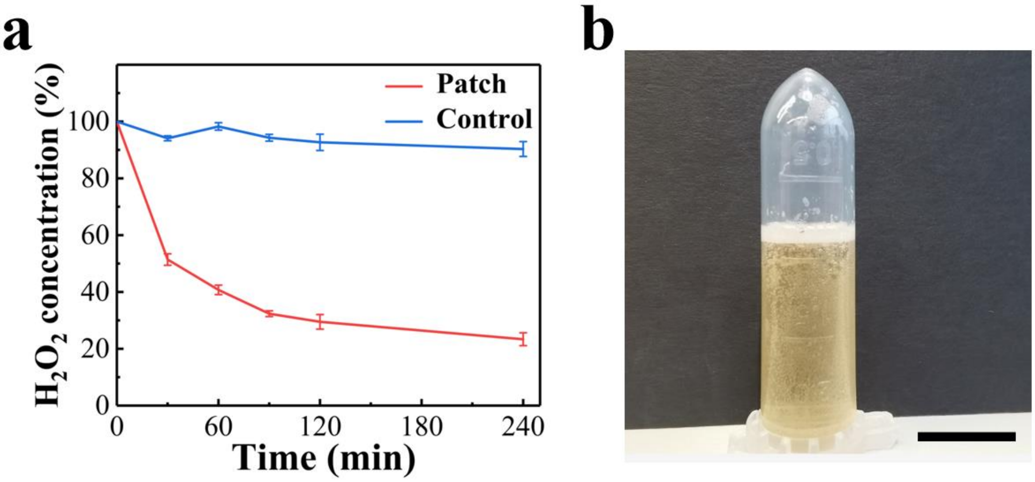

Figure S16. (a) The ROS scavenging efficiency of the patch. Concentration of H_2O_2 was 10 mmol/L. (b) Qualitative experiment of ROS scavenging. After the addition of mucus, the 1% H_2O_2 solution decomposed to produce oxygen, proving its catalytic decomposition ability. Scale bar is 1 cm. For (a), data are shown as mean $\pm$ SD, n = 3.
